# Thymine functionalised porphyrins, synthesis and heteromolecular surface-based self-assembly†
†Electronic supplementary information (ESI) available: Additional experimental details including: full synthetic methods and characterization; full details of single crystal X-ray structure refinement and CIFs; binding measurements; STM experiments; and MM simulations. CCDC 1034260 and 1034261. For ESI and crystallographic data in CIF or other electronic format see DOI: 10.1039/c4sc03531c



**DOI:** 10.1039/c4sc03531c

**Published:** 2014-12-11

**Authors:** Anna G. Slater, Ya Hu, Lixu Yang, Stephen P. Argent, William Lewis, Matthew O. Blunt, Neil R. Champness

**Affiliations:** a School of Chemistry , University of Nottingham , University Park , Nottingham , NG7 2RD UK . Email: Neil.Champness@nottingham.ac.uk; b The Department of Chemistry , University College London (UCL) , London , WC1H 0AJ , UK . Email: m.blunt@ucl.ac.uk

## Abstract

The synthesis and surface-based self-assembly of thymine-functionalised porphyrins is described. Reaction of 1-formylphenyl-3-benzoyl-thymine with suitable pyrollic species leads to the formation of tetra-(phenylthymine)porphyrin (tetra-TP) or mono-thymine-tri-(3,5-di-*tert*-butylphenyl)porphyrin (mono-TP). Single crystal X-ray diffraction studies demonstrate the self-association of mono-TP in the solid state through thymine···thymine hydrogen-bonding interactions but in solution this interaction (*K*_d_ = 6.1 ± 3.0 M^–1^) is relatively weak in comparison to the heteromolecular interaction between mono-TP and 9-propyladenine (*K* = 91.8 ± 20.5 M^–1^). STM studies of the tetratopic hydrogen-bonding tecton, tetra-TP, deposited on an HOPG substrate reveal the formation of an almost perfectly square self-assembled lattice through thymine···thymine hydrogen-bonding. Co-deposition of tetra-TP with 9-propyladenine leads to the adoption of preferable thymine···adenine interactions leading to the formation of a heteromolecular tetra-TP···9-propyladenine hydrogen bonded array including both Watson–Crick thymine···adenine interactions and adenine···adenine hydrogen-bonding. The studies demonstrate a pathway for the self-assembly of tetratopic hydrogen-bonding tectons and the use of preferential heteromolecular thymine···adenine interactions for the disruption of the homomolecular tetra-TP array. Studies of the self-assembly of tetra-TP and 9-propyladenine demonstrate a strong dependence on overall concentration and molar ratio of components indicating the importance of kinetic effects in surface self-assembly processes.

## Abstract 2

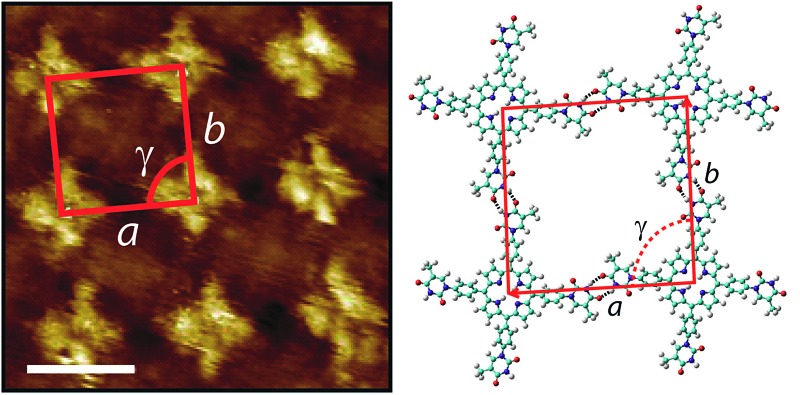
The synthesis and surface-based self-assembly of thymine-functionalised porphyrins is described.

## Introduction

The study of self-assembled two-dimensional structures on surfaces has become an area of intense interest over recent years.^1–5^ Particular focus has been applied to the use of intermolecular interactions in attempts to control the relative organisation of molecules and to create well-defined molecular arrangements. Intermolecular interactions that have been successfully used to prepare self-assembled structures include coordination bonds,^6,7^ halogen bonds^8,9^ and van der Waals interactions.^10–12^ Hydrogen-bonds^13–21^ have received extensive attention with a major focus being the exploitation of molecules bearing multiple carboxylic acids to form extended arrays.^13–18^ It is also possible to prepare surface-based self-assembled structures from more than one molecular component^22^ using the well-established concepts within supramolecular chemistry of the molecular tecton^23,24^ and supramolecular synthon.^25^ By using two or more tectons that are designed such that they incorporate hydrogen-bonding moieties that favour heteromolecular interactions multi-component arrays can be targeted.^19–21^

Perhaps the most widely known heteromolecular synthons are those formed between DNA nucleobases. Nucleobases have been extensively studied in the field of surface-based self-assembly with a particular focus on understanding their hydrogen-bonding behaviour. Two recent reviews cover advances in the area^26,27^ and illustrate the complexity of such systems. Nucleobases have been studied using scanning tunnelling microscopy^28–31^ including thymine (T)^30^ and adenine (A),^31^ of particular relevance to this study. Throughout these studies a variety of substrates have been used, including metallic surfaces and highly-ordered pyrolitic graphite (HOPG), as well as a variety of conditions, ultra-high vacuum and solid–liquid interfaces. A study of particular interest is that by Besenbacher *et al.* who describe investigations of combinations of thymine and adenine.^32^ As anticipated intermolecular hydrogen-bonding interactions are observed between the thymine and adenine generating A–T–A–T quartets that involve reverse Hoogsteen hydrogen-bonding. Examples of functionalized adenine and thymine molecules have been studied in attempts to influence the nature of the intermolecular hydrogen-bonding interactions. Notable examples, described by Bonifazi and co-workers,^33,34^ report the synthesis of di-uracil rods and their subsequent self-assembly with molecules that present hydrogen-bonding motifs complementary to that of uracil (and thymine).

**Scheme 1:**
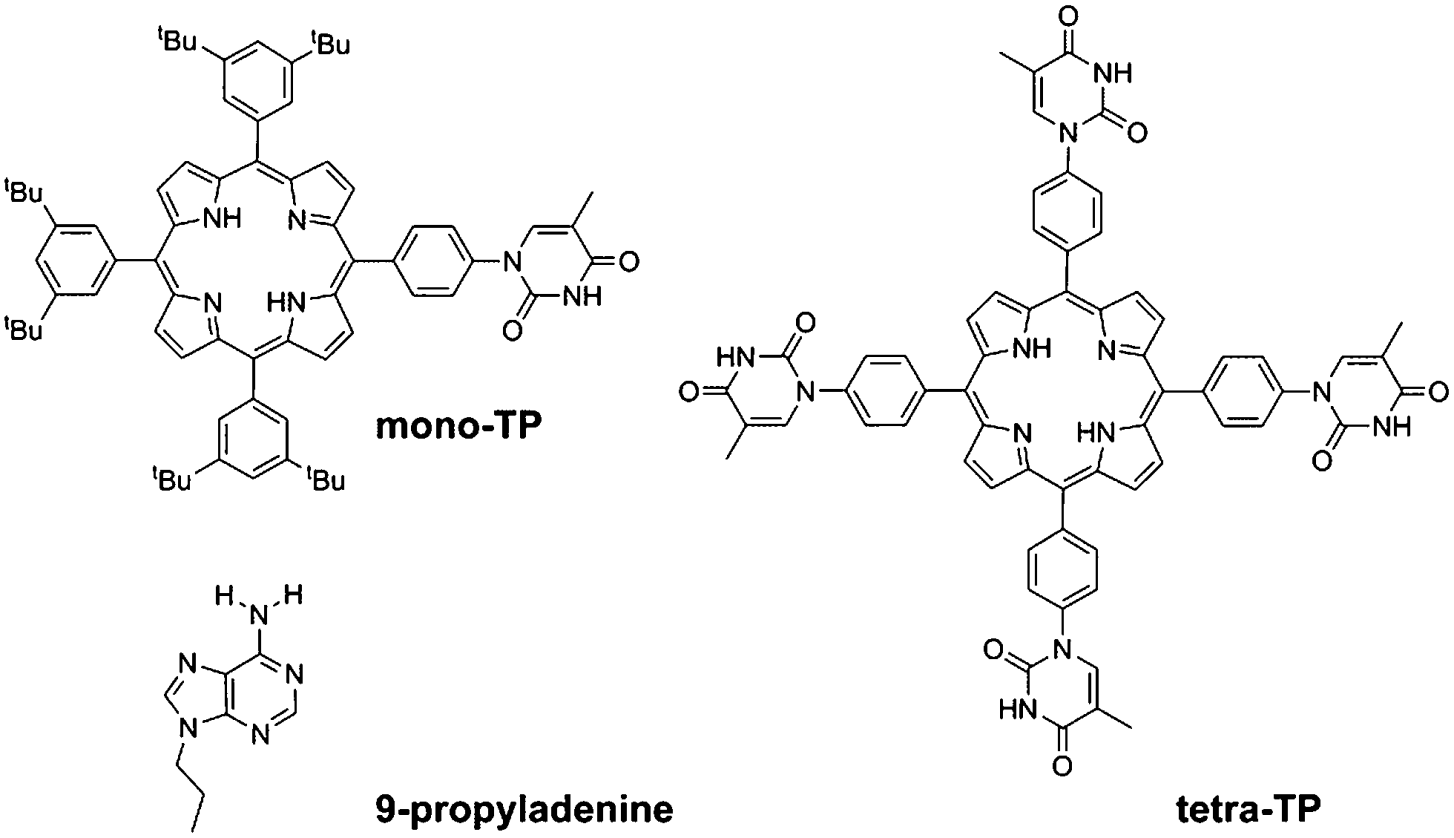
Molecules used in this study.

In this study we have synthesised thymine-functionalised porphyrin molecules, or tectons, (Scheme 1) that present the imide hydrogen bonding moiety of thymine in such a manner to encourage divergent assembly with either other molecules of the same type or with a simple propyl-functionalised adenine species, 9-propyl-9*H*-purine-6-ylamine (9-propyladenine). Porphyrins represent useful scaffolds for the basis of hydrogen-bonding tectons as functionalization of each *meso*-position leads to a tetratopic tecton. The vast majority of tectons previously investigated in surface self-assembled networks^1–5^ are either rod-like ditopic tectons^6,13,19–21^ or tritopic^7,12,14,19–21^ tectons. Although tetratopic hydrogen-bonding tectons are known, notably with carboxylic acid groups,^15–18^ systems with more specific hydrogen-bonding moieties for heteromolecular arrays have not been studied previously for surface self-assembly. Porphyrins present a highly attractive target for the basis of tetratopic tectons due to their fourfold symmetry and have been used for the assembly of a variety of surface-based self-assembled structures^35^ including covalently-coupled arrays^36^ and, notably with carboxylic acid hydrogen-bonding moieties through the use of 5,10,15,20-tetrakis-(4-carboxylphenyl)-porphyrin.^37^

Two target porphyrin molecules were identified: tetra-(phenylthymine)porphyrin (tetra-TP) for surface-based self-assembly studies and mono-thymine-tri-(3,5-di-*tert*-butylphenyl)porphyrin (mono-TP) as a model compound to probe the nature of intermolecular interactions in the solution phase. The basic design encompassed functionalisation of the porphyrin core with phenylthymine groups and the remaining *meso* positions, in the case of mono-TP, bearing 3,5-di-*tert*-butylphenyl moieties. The *tert*-butyl functionalised appendages were chosen due to their ability to enhance porphyrin solubility, inhibiting π–π interactions, and allowing more facile solution-based studies. The synthesis of a porphyrin functionalised with uracil in the *meso*-position, 10,15,20-tetrakis(1-butyl-6-uracyl)porphyrin, has been reported previously^38^ and was found to form nanofibre structures. The design of 10,15,20-tetrakis(1-butyl-6-uracyl)porphyrin is not appropriate for surface self-assembly studies due to the orthogonal arrangement of the uracil moiety with respect to the porphyrin core. Thus we adopted a design that positioned a phenyl group between the porphyrin plane and the thymine group so that both porphyrin and thymine could be co-planar and parallel to the surface that the molecules are to be deposited upon. Our studies demonstrate that it is not only possible to prepare homomolecular hydrogen-bonded arrays with tetra-TP but heteromolecular arrays can be generated by combination of suitable tectons, in our case tetra-TP and 9-propyladenine.

## Results and discussion

### Synthesis and structural studies

Porphyrin molecules were prepared functionalised in the *meso*-positions by phenyl–thymine moieties. Our approach required the synthesis of 1-formylphenyl-3-benzoyl-thymine from 4-formylphenylboronic acid and 3-benzoylthymine^39^ using a Cu(OAc)_2_-mediated Chan–Lam–Evans-modified Ullmann condensation reaction to facilitate the cross-coupling process.^40^ Reaction of 1-formylphenyl-3-benzoyl-thymine with a large excess of pyrrole in the presence of InCl_3_ affords the desired dipyrromethane which can be subsequently used in porphyrin synthesis.

**Scheme 2:**
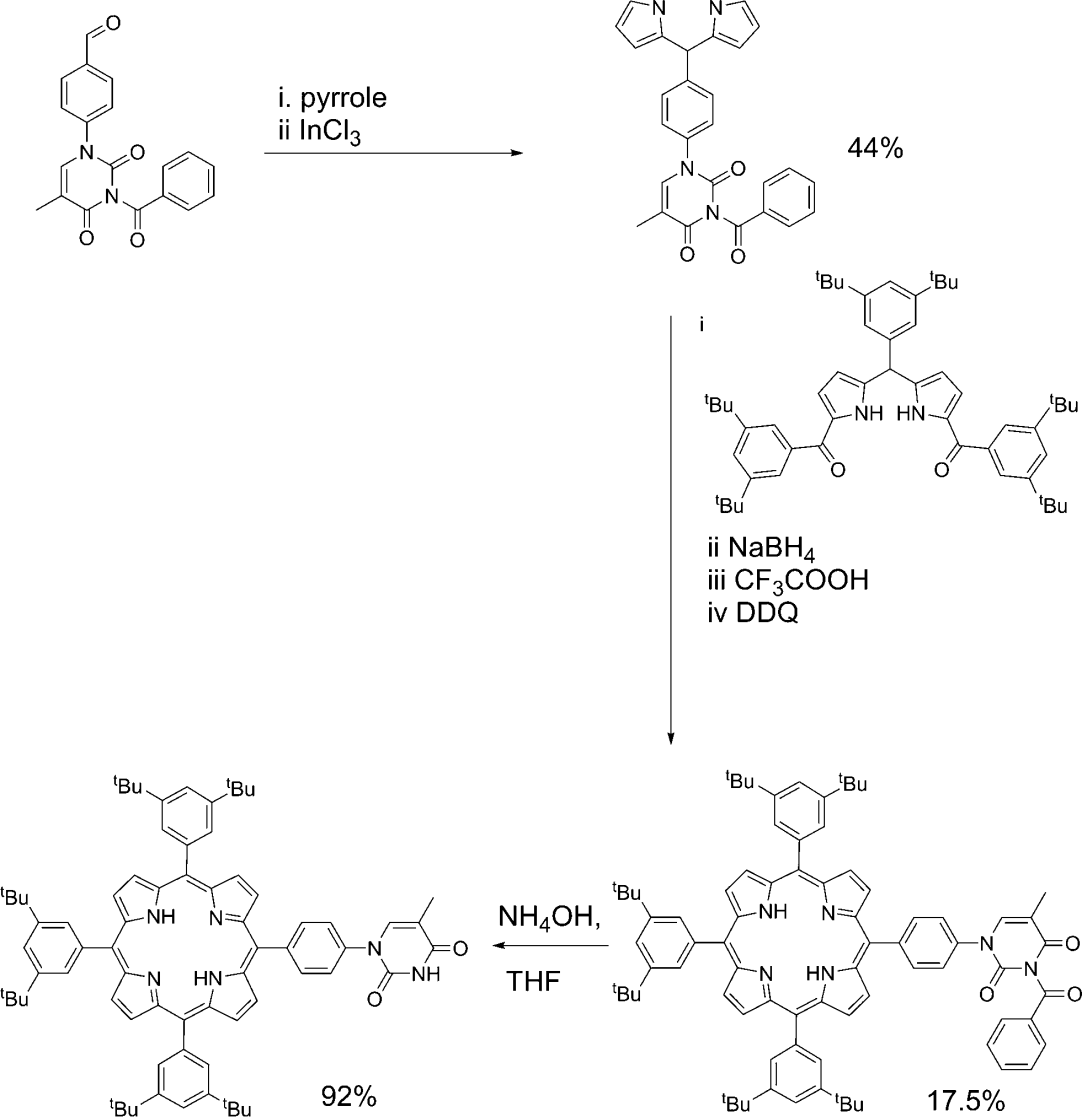
Synthetic route used for the preparation of mono-TP.

**Scheme 3:**
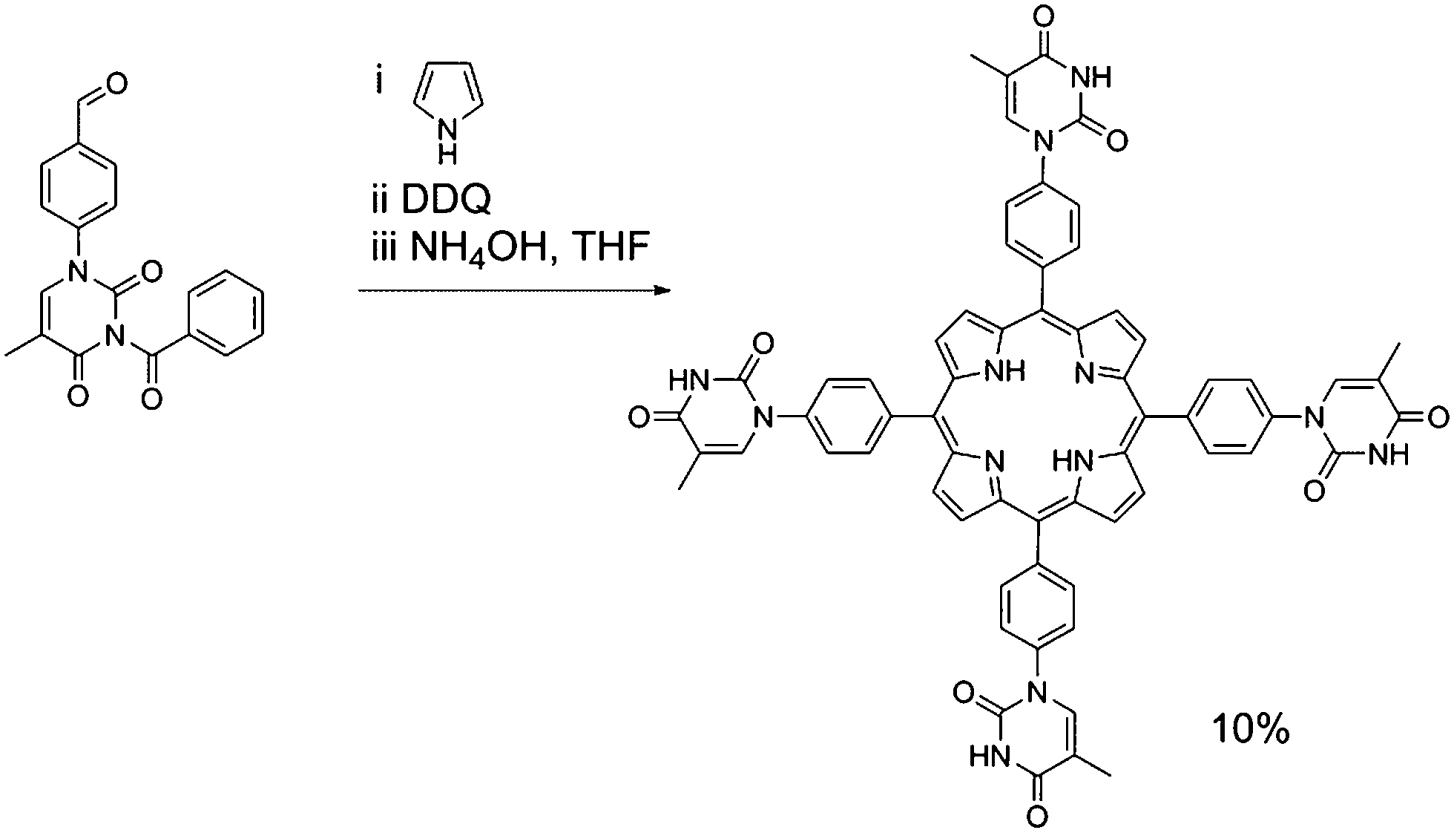
Synthetic route used for the preparation of tetra-TP.

In the case of mono-TP (Scheme 2) the benzoyl-thymine functionalised dipyrromethane was reacted with a suitable *tert*-butyl functionalised carbinol species, using Lindsey's approach,^41^ in the presence of trifluoroacetic acid (TFA) with subsequent oxidation using 2,3-dichloro-5,6-dicyano-1,4-benzoquinone (DDQ). Subsequent deprotection of the benzoyl-protected thymine was achieved in good yield using NH_4_OH as the deprotecting agent.^42^ The synthesis of the tetra-TP was more straightforward (Scheme 3) following direct reaction of 1-formylphenyl-3-benzoyl-thymine with a large excess of pyrrole using TFA to initiate the reaction. Oxidation with DDQ gave the target molecule in 11% yield and deprotection, again using NH_4_OH,^42^ afforded tetra-TP in 91% yield.

**Fig. 1:**
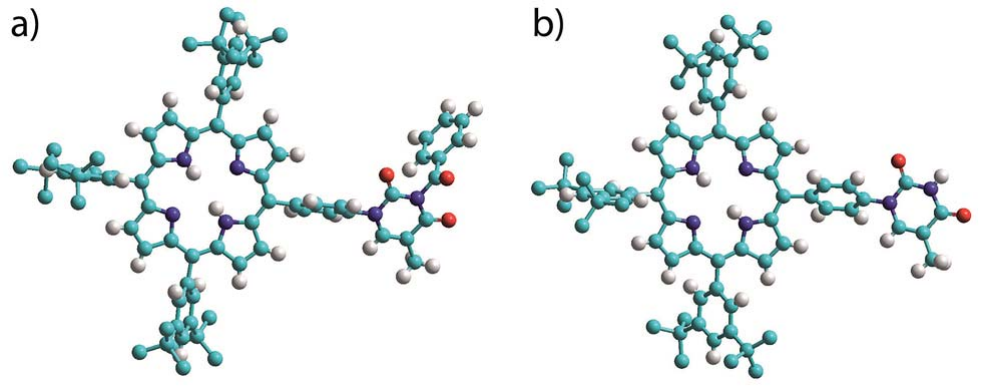
Single crystal X-ray structures of (a) benzoyl-mono-TP and (b) mono-TP. Note the relative orientation of the thymine moiety with respect to the porphyrin plane. Hydrogen atoms of the *tert*-butyl groups are omitted for clarity. Atoms are colored as follows C – light blue; N – dark blue; H – white; O – red.

The products were characterised by conventional techniques and in the case of mono-TP and its benzoyl-protected precursor by single crystal X-ray diffraction. For both of these compounds slow diffusion of MeOH into a solution of the compound, either CH_2_Cl_2_ (mono-TP) or CDCl_3_ (benzoyl-mono-TP), led to growth of single crystals of suitable quality for X-ray diffraction studies. In both cases the X-ray structure confirms the identity of the product and the relative arrangement of the thymine moiety with respect to the porphyrin ring (Fig. 1).

**Fig. 2:**
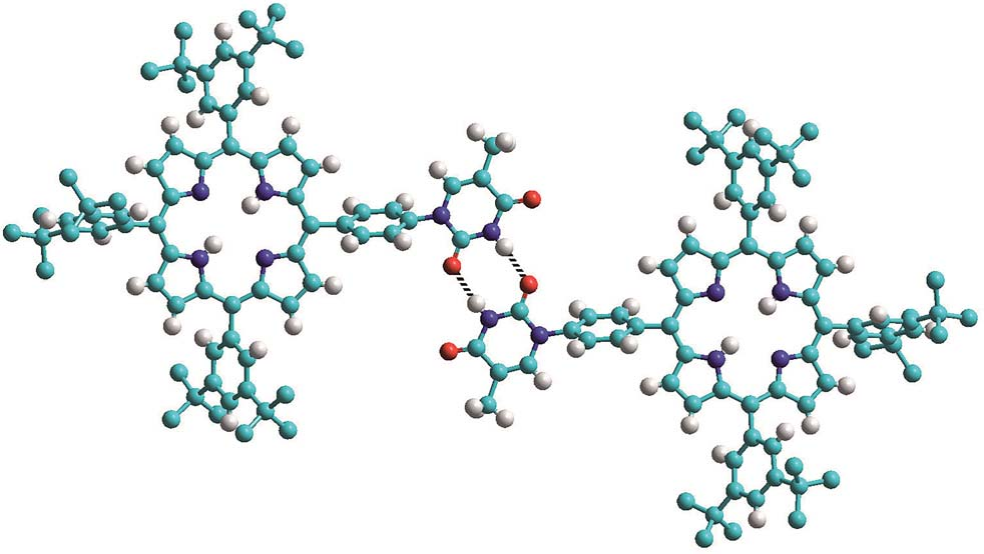
View of the single crystal X-ray structure of mono-TP illustrating the formation of the inter-thymine R_2_^2^(8) double hydrogen-bonding interaction (represented with the two dotted lines) observed in the solid state. Hydrogen atoms of the *tert*-butyl groups are omitted for clarity. Atoms are colored as follows C – light blue; N – dark blue; H – white; O – red.

The structure of the mono-TP reveals valuable information about the nature of the intermolecular interactions anticipated for thymine-substituted porphyrin species (Fig. 2). The most pertinent feature is the formation of an R_2_^2^(8) double hydrogen-bonding interaction^43^ between thymine moieties on adjacent molecules. Each N–H···O hydrogen bond within the inter-thymine R_2_^2^(8) synthon is crystallographically equivalent [N···O = 2.818(3) Å; H···O = 1.94 Å; ∠N–H···O = 172.0°] and falls within the typical range expected for such a hydrogen bond.^44^ Adjacent molecules pack such that π–π interactions are observed between the thymine moiety of one molecule and a pyrrole of an adjacent molecule (centroid···centroid separation of 3.50 Å). As anticipated in both structures, benzoyl-mono-TP and mono-TP, the phenyl ring that links the porphyrin moiety with the thymine group adopts an orientation that approaches orthogonality with respect to the porphyrin plane (71.02°: benzoyl-mono-TP; 64.01°: mono-TP) and the thymine approaches co-planarity with the porphyrin core in both instances (17.21°: benzoyl-mono-TP; 13.37°: mono-TP).

**Fig. 3:**
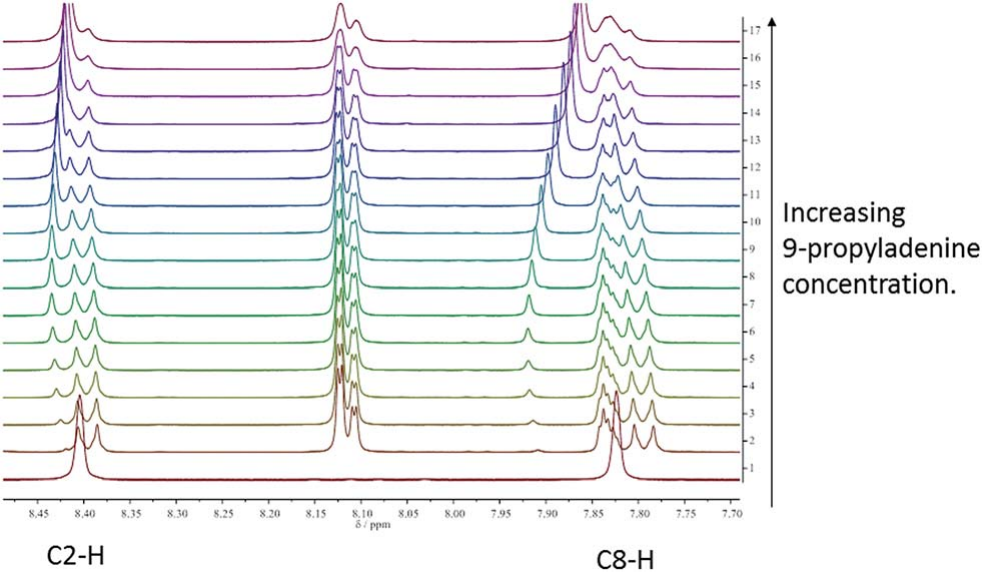
^1^H NMR spectrum illustrating shifts in the C8–H and C2–H proton signals during titration of mono-TP with 9-propyladenine. Spectrum 1 corresponds to pure 9-propyladenine and spectra 2–17 are recorded with constant mono-TP concentration and increasing 9-propyladenine concentration.

In order to assess the strength of interaction between porphyrin-appended thymine moieties and a simple propyl-functionalised adenine species, 9-propyl-9*H*-purine-6-ylamine (9-propyladenine), a series of binding studies were undertaken using solutions of mono-TP in CDCl_3_ solution,^45^ see ESI† for details (tetra-TP was found to be have insufficient solubility in suitable solvents to allow binding studies). The possibility of self-association needs to be considered, particularly considering the observed thymine···thymine interactions in the single crystal structure of mono-TP. Thus self-association binding constants were measured for mono-TP (*K*_d_ = 6.1 ± 3.0 M^–1^) and 9-propyladenine (*K*_d_ = 2.8 ± 1.7 M^–1^) and found to be small in both instances. In contrast the binding constant for the interaction between mono-TP and 9-propyladenine was found to be *K* = 91.8 ± 20.5 M^–1^ indicating a favourable hetero-intermolecular interaction between the two species as anticipated. The NMR studies also indicate both Hoogsteen^46^ and Watson–Crick^47^ binding modes between the thymine and adenine moieties with corresponding shifts in the C8–H and C2–H proton signals respectively (Fig. 3).

### Surface self-assembly studies

The surface self-assembly of tetra-TP and 9-propyladenine was investigated at liquid–solid interfaces between highly oriented pyrolytic graphite (HOPG) and an organic solvent layer using scanning tunnelling microscopy (STM). All attempts to adsorb mono-TP onto HOPG were unsuccessful which we attribute to weaker adsorption of this molecule on this surface under the conditions used, in part due to the enhanced solubility of this molecule in comparison to tetra-TP and the smaller number of potential intermolecular hydrogen-bonding interactions.

Mixtures of tetrahydrofuran (THF) and 1,2,4-trichlorobenzene (TCB) with a 1 : 9 volume ratio respectively were used as solvents for the tetra-TP and 9-propyladenine. Solutions in the concentration range 5 × 10^–6^ to 5 × 10^–5^ M for tetra-TP and 8 × 10^–6^ to 1 × 10^–3^ M for the 9-propyladenine were employed. Even at these low concentrations the tetra-TP solutions were saturated with some visible undissolved material. It was found that saturated solutions were required in order to form self-assembled surface structures. A small quantity of saturated solution was deposited on to a preheated HOPG substrate held at 60 °C. After 5 minutes held at this temperature the substrate was allowed to cool to room temperature before STM imaging.

**Fig. 4:**
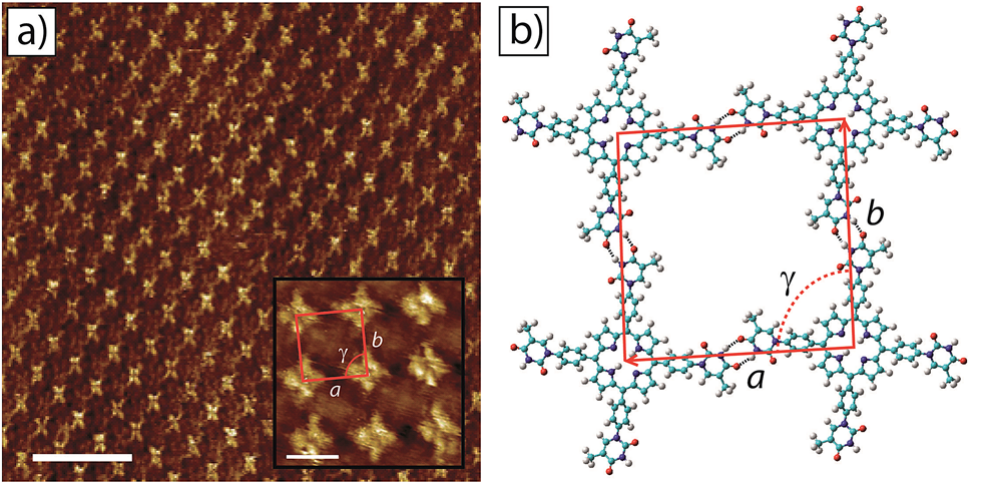
2D self-assembled network of tetra-TP at the TCB–HOPG liquid–solid interface. (a) Large scale STM image of the tetra-T-porphyrin (2.82 × 10^–5^ M) network at the TCB–HOPG liquid–solid interface. The insert shows a high resolution, drift corrected STM image of the network with an individual 2D unit cell marked in red: unit cell parameters *a* = 25.9 ± 0.5 Å; *b* = 25.2 ± 0.6 Å; and *γ* = 91 ± 1°. STM imaging parameters: *V*_s_ = –0.5 V; *I*_t_ = 15 pA. Scale bar = 20 nm (insert = 2 nm). (b) Molecular model of the tetra-TP network from MM simulations. Unit cell parameters *a* = 26.0 Å; *b* = 25.9 Å; and *γ* = 90°.

STM images of tetra-TP self-assembled structures clearly display the cruciform shape and symmetry of the tetra-TP molecules suggesting they adsorb in a planar fashion on the HOPG substrate (Fig. 4a). The self-assembled structure displays large domains (>100 nm) of a hydrogen bond stabilised network with a P2 plane symmetry group. Analysis of drift corrected STM images (Fig. 4a insert) provide 2D unit cell dimensions for this structure of *a* = (25.9 ± 0.5) Å; *b* = (25.2 ± 0.6) Å and *γ* = (90 ± 2)° and angles between the unit cell vectors and underlying HOPG symmetry axes of *α* = (6 ± 3)°; and *β* = (25 ± 3)°. For details of the drift correction process applied to the STM images see the ESI.†

Fig. 4b shows a structural model derived from molecular mechanics (MM) simulations for the tetra-TP network. This molecular structure is stabilised by thymine–thymine hydrogen bond dimers formed between adjacent tetra-TP molecules, in a similar fashion to the bonding arrangement observed in the single crystal structure of mono-TP. The inter-thymine hydrogen-bonding arrangement is also similar to that observed for thymine adsorbed at the liquid–solid interface between 1-octanol and HOPG.^32^ Additionally tetrakis(4-carboxy-phenyl)-porphyrin molecules adopt a similar hydrogen bond stabilised structure on an Au(111) surface^37*c*^ where the network is stabilised *via* carboxylic acid hydrogen bond dimers. The MM simulations were carried out with the molecules placed above a single fixed layer of graphene; further details of the simulations are available in the ESI.† Unit cell dimensions and measured from geometry optimised structures produced values of *a* = 26.0 Å; *b* = 25.9 Å and *γ* = 91° and angles between the unit cell vectors and underlying graphite symmetry axes of *α* = 4.7°; and *β* = 24.3°, in good agreement with the experimentally determined values.

The asymmetric nature of the thymine groups decorating the tetra-TP molecules indicates that the molecules have the potential to adopt a chiral arrangement when adsorbed onto a surface.^48^ Numerous previous studies have shown that prochiral molecules tend to assemble into homochiral domains on surfaces containing molecules of only a single handedness.^49^ The almost perfectly square 2D unit cell observed for the tetra-TP structure suggests that all of the thymine groups within an individual tetra-TP molecule display the same orientation with respect to the porphyrin core, and that individual domains contain only molecules of a single handedness. The overall surface structure of tetra-TP remains globally achiral by forming an equal area of mirror domains containing either right, or left-handed molecules. STM images showing the mirror image tetra-TP domain to that observed in Fig. 4 are shown in the ESI.† There is no significant barrier to rotation of the thymine groups prior to surface adsorption and although we see no evidence of porphyrin molecules with mixed thymine orientations we cannot rule out the possibility of such orientations existing in small localised regions.

**Fig. 5:**
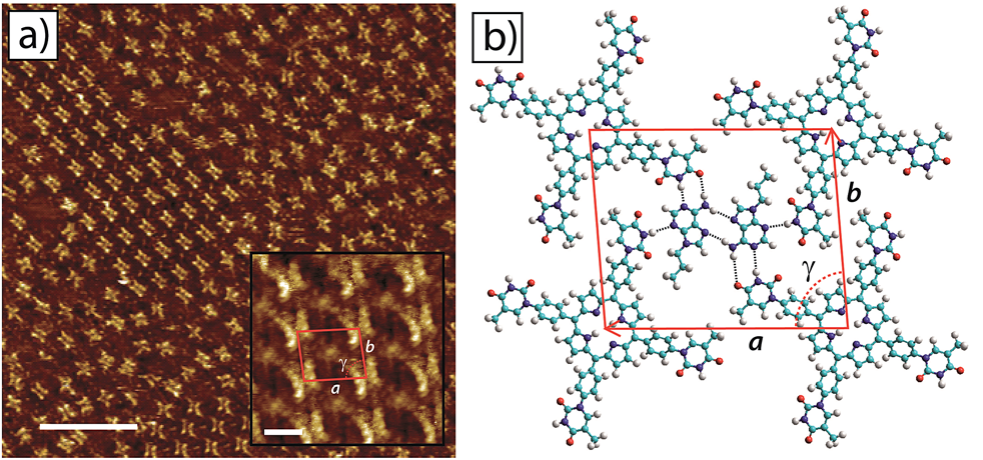
2D self-assembled network of tetra-TP and 9-propyladenine at the TCB–HOPG liquid–solid interface. (a) Large scale STM image of the tetra-TP (2.82 × 10^–5^ M) and 9-propyladenine (4.52 × 10^–4^ M) network at the TCB–HOPG liquid–solid interface. The insert shows a high resolution, drift corrected STM image of the network with an individual 2D unit cell marked in red: unit cell parameters from STM images *a* = 25.4 ± 0.7 Å; *b* = 22.2 ± 0.9 Å; and *γ* = 83 ± 2°. STM imaging parameters: *V*_s_ = –0.5 V; *I*_t_ = 15 pA. Scale bar = 200 Å (insert = 16 Å). (b) Molecular model of the tetra-T-porphyrin network from MM simulations. Unit cell parameters from MM simulations *a* = 26.0 Å; *b* = 21.5 Å; and *γ* = 85.7°.

The surface self-assembly of mixtures of tetra-TP and 9-propyladenine on HOPG produced a molecular network with a combination of disordered regions interspersed with small domains of an ordered co-crystal structure containing both tetra-TP and 9-propyladenine. Fig. 5a shows a large scale STM image displaying both the disordered structure (top right hand corner) and the ordered co-crystal (top left hand corner). Analysis of high resolution drift corrected STM images for the tetra-TP–9-propyladenine co-crystal (Fig. 5a insert) produce unit cell dimensions of *a* = (25.4 ± 0.7) Å; *b* = (22.2 ± 0.9) Å and *γ* = (83.0 ± 2.0)° and angles between the unit cell vectors and underlying HOPG symmetry axes of *α* = (4.4 ± 1.2)°; and *β* = (26.9 ± 2.1)°.

Using the same method as adopted for the tetra-TP network MM simulations were performed to produce a molecular model for the tetra-TP and 9-propyladenine co-crystal network (Fig. 5b). This structure consists of a pair of 9-propyladenine molecules surrounded by 4 tetra-TP molecules. The 9-propyladenine molecules are linked to each other in a dimeric hydrogen bonded arrangement and each 9-propyladenine is further linked to the surrounding tetra-TP molecules by three hydrogen bonds. The dimeric arrangement of the two 9-propyladenine molecules is the same as that calculated to be the most stable arrangement and previously observed for unfunctionalised adenine adsorbed on HOPG.^32^ Two of the hydrogen bonds linking the 9-propyladenine to one of the neighbouring tetra-TP molecules adopt a Watson–Crick^47^ binding mode. The remaining available hydrogen-bonded site on 9-propyladenine, the N3 position, adopts a conventional N–H···N hydrogen bond to a further tetra-TP molecule. Unit cell dimensions measured from geometry optimised MM simulations gave values of *a* = 26.0 Å; *b* = 21.5 Å; and *γ* = 85.7°. The angles between the unit cell vectors and underlying graphite symmetry axes were *α* = 4.7°; and *β* = 30.0°, in good agreement with the experimentally determined values. Similarly to the tetra-TP network, the tetra-TP and 9-propyladenine co-crystal also displays the formation of homochiral domains in which the thymine groups of the chiral tetra-TP molecule all adopt the same orientation with respect to the porphyrin core. An STM image displaying two mirror image domains of the tetra-TP and 9-propyladenine co-crystal is provided in the ESI.†

**Fig. 6:**
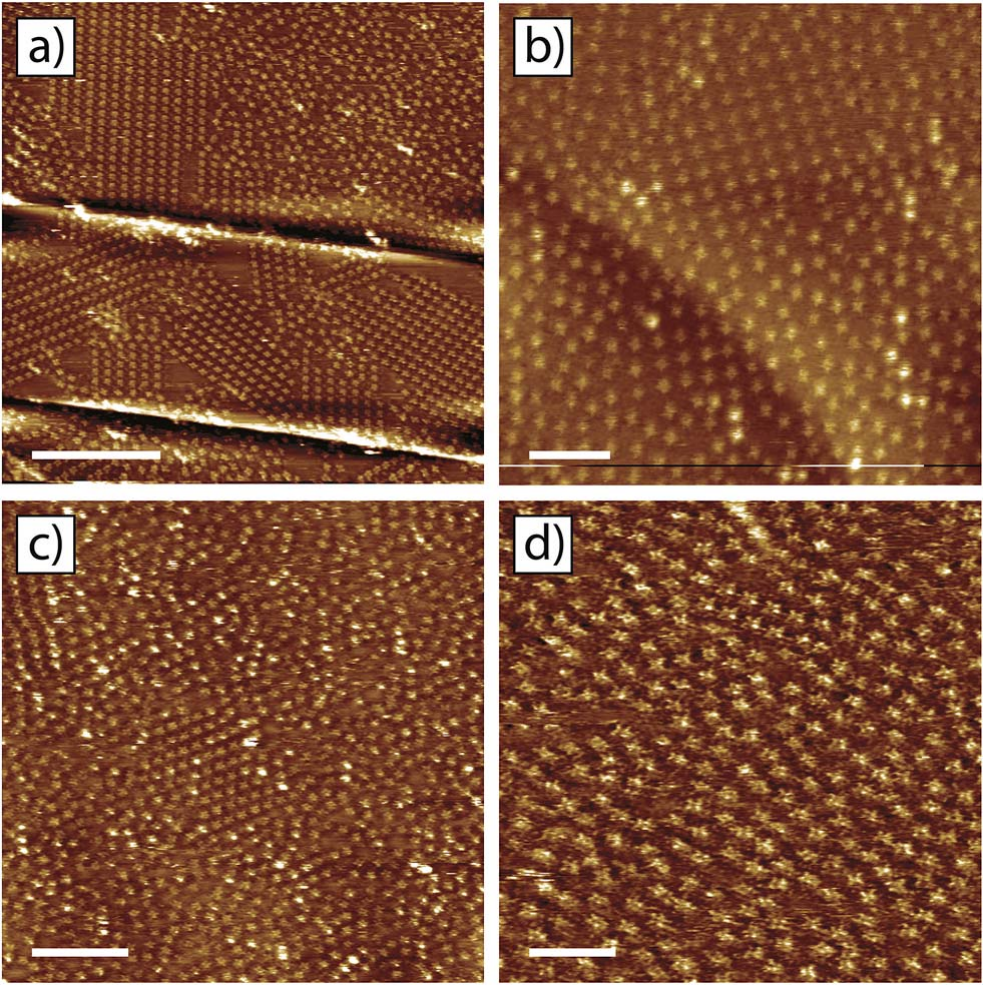
Influence of the concentration and molar ratio of components on the morphology of the tetra-TP–9-propyladenine network. (a) Tetra-TP (1.41 × 10^–5^ M) and 9-propyladenine (2.26 × 10^–4^ M) mixture: molar ratio 1 : 16. (b) Tetra-TP (3.03 × 10^–5^ M) and 9-propyladenine (8.07 × 10^–6^ M) mixture: molar ratio 4 : 1. (c) Tetra-TP (1.77 × 10^–5^ M) and 9-propyladenine (1.13 × 10^–3^ M) mixture: molar ratio 1 : 64. (d) Tetra-T-porphyrin (5.04 × 10^–6^ M) and 9-propyladenine (1.93 × 10^–3^ M) mixture: molar ratio 1 : 383. STM imaging parameters: *V*_s_ = –0.5 V; *I*_t_ = 15 pA. Scale bars: (a) 40 nm; (b) 10 nm; (c) 20 nm; (d) 10 nm.

The domain size for the tetra-TP and 9-propyladenine co-crystal and the prevalence of the disordered structure are linked to both the concentration and ratio of the individual components. Fig. 6a–d shows four example STM images of structures produced using different concentrations and molar ratios of tetra-TP and 9-propyladenine. Fig. 6a has the same molar ratio of tetra-TP to 9-propyladenine, 1 : 16, as Fig. 5a but half the overall molar concentration. The domain size for the tetra-TP and 9-propyladenine co-crystal has increased at the expense of the disordered arrangement.

Fig. 6b has the same molar concentration of tetra-TP as Fig. 5a but a greatly reduced concentration of 9-propyladenine so that in this case the molar ratio of tetra-TP to 9-propyladenine is 4 : 1. In this case the tetra-TP and 9-propyladenine co-crystal structure is not present; instead we observe the disordered network interspersed with domains of the tetra-TP mono-component network. As the amount of 9-propyladenine in the mixture is increased domains of the tetra-TP and 9-propyladenine co-crystal reduce in size until we are left with primarily the disordered structure. This effect can be seen in Fig. 6c and d which show example STM images of mixtures with molar ratios of tetra-TP to 9-propyladenine of 1 : 64 and 1 : 383 respectively. The delicate dependence of the co-crystal structure on the concentration and molar ratio of the components suggests that kinetic effects play a critical role in the self-assembly of tetra-TP and 9-propyladenine.

## Conclusions

We have demonstrated a successful strategy for employing tetratopic tectons in the preparation of surface-based self-assembled arrays. Inter-thymine hydrogen bonds successfully lead to the formation of square-grid lattices upon deposition of tetra-TP onto an HOPG substrate. The preferential formation of heteromolecular hydrogen-bonds exhibited by thymine and adenine decorated tectons, validated in solution by NMR studies, can be exploited to disrupt the homomolecular tetra-TP array and leads to the formation of a co-crystal between tetra-TP and 9-propyladenine. Studies of the self-assembly of tetra-TP and 9-propyladenine demonstrate a strong dependence on overall concentration and molar ratio of components. Lower overall concentrations lead to a greater proportion of ordered domains and the degree of order in the resulting structures is highly dependent on the molar ratio of tetra-TP : 9-propyladenine. These studies indicate the importance of kinetic effects in surface self-assembly processes.

The system reported herein illustrates a successful strategy that has wide implications for the design of new tectons to be used in the formation of heteromolecular arrays. We demonstrate that the exploitation of the familiar recognition pathways of DNA bases can be incorporated into complex molecules and used to generate heteromolecular arrays. This approach has far-reaching implications for molecule/tecton design for preparing specific hydrogen-bonded arrays on surfaces and strongly indicates the possibility of preparing complex multicomponent arrays using a self-assembly stratagem.

## Experimental

Details of the synthetic procedures employed are described in the ESI.† The following intermediate species were synthesised following literature methods: 3,5-di-*tert*-butylbenzoic acid,^50^ 3,5-di-*tert-*butylbenzaldehyde,^51^ 3,5-di*-tert*-butylphenyl-dipyrromethane,^52^ 3-benzoylthymine^39^ and 9-propyladenine.^53^

CCDC-1034260 (mono-TP) and CCDC-; 1034261 (benzoyl-mono-TP) contain the ESI† for this paper. Further details of the single crystal structure refinements are given in ESI† but details of the final refinement parameters are as follows for the structures of mono-TP and benzoyl-mono-TP.

### Crystal data for mono-TP

C_74_H_86_N_6_O_3_ (*M* = 1107.48): triclinic, space group *P*1 (no. 2), *a* = 9.8270(6) Å, *b* = 16.9659(8) Å, *c* = 21.6298(13) Å, *α* = 69.273(5)°, *β* = 81.505(5)°, *γ* = 87.976(5)°, *V* = 3335.2(4) Å^3^, *Z* = 2, *T* = 120(2) K, *μ*(synchrotron) = 0.063 mm^–1^, *D*_calc_ = 1.103 g mm^–3^, 31 197 reflections measured, 11 500 unique (*R*_int_ = 0.0549) which were used in all calculations. The final *R*_1_ was 0.0756 (*I* > 2*σ*(*I*)) and w*R*_2_ was 0.2097 (all data).

### Crystal data for mono-phenyl(benzoylthymine)-tri-(3,5-di-*tert*-butylphenyl)porphyrin 6CHCl_3_

C_86_H_92_Cl_18_N_6_O_3_ (*M* = 1895.75): triclinic, space group *P*1 (no. 2), *a* = 15.4721(5) Å, *b* = 16.3717(5) Å, *c* = 19.0278(6) Å, *α* = 92.068(2) °, *β* = 110.731(3) °, *γ* = 99.280(3)°, *V* = 4426.0(3) Å^3^, *Z* = 2, *T* = 120(2) K, *μ*(Cu-Kα) = 0.063 mm^–1^, *D*_calc_ = 1.422 g mm^–3^, 52 160 reflections measured, 15 638 unique (*R*_int_ = 0.079) which were used in all calculations. The final *R*_1_ was 0.1220 (*I* > 2*σ*(*I*)) and w*R*_2_ was 0.3846 (all data).

Details of the STM experiments, image analysis and MM simulations can be found in the ESI.†
