# Supplementary material for: Thymine functionalised porphyrins, synthesis and heteromolecular surface-based self-assembly
Source: Chem Sci. 2014 Dec 11;6(2):1562–9. doi: 10.1039/c4sc03531c (PMC5811115; doi:10.1039/c4sc03531c)
Supplement: Supplementary file 1 [file SC-006-C4SC03531C-s001.pdf]

Electronic Supplementary Material (ESI) for Chemical Science.
This journal is © The Royal Society of Chemistry 2014

Supporting Information: *Thymine Functionalised Porphyrins, Synthesis and Surface-based Self-assembly.* Anna G. Slater, Ya Hu, Lixu Yang, Stephen P. Argent, William Lewis, Matthew O. Blunt and Neil R. Champness

Experimental:

Synthetic Procedures. 3,5-di-*tert*-butylbenzoic acid,[1] 3,5-di-*tert*-butylbenzaldehyde,[2] 3,5-di-*tert*-butylphenyl-dipyrromethane,[3] 3-benzoylthymine[4] and 9-propyladenine[5] were synthesised according to literature procedures.

3,5-di-tert-butylphenyl morpholin-4-yl ketone. 3,5-di-*tert*-butylbenzoic acid (7 g, 29.9 mmol) was added to a flame dried flask which had been backfilled with nitrogen three times. DCM (70 mL) was added *via* canula, followed by DMF (0.77 mL, 9.9 mmol) and oxalyl chloride (2.3 mL, 26.9 mmol) added dropwise over 15 minutes. The mixture was stirred at room temperature for 1 h 30 under a nitrogen atmosphere. In a second flame dried, backfilled flask, morpholine (9.3 mL, 108 mmol) and triethylamine (4.3 mL, 30.8 mmol) was added to DCM (70 mL). This mixture was added to the first flask slowly via canula over 20 minutes. The solution was stirred at room temperature for 2 h under a nitrogen atmosphere. The mixture was then quenched with 0.5 M HCl (100 mL) and stirred for 30 minutes at room temperature. The organic layer was then separated, washed with water (3 × 100 mL), dried over $MgSO_4$ and evaporated under reduced pressure to yield pure compound (8.98 g, 99 %). MS (EI): m/z^+ Calc = 304.2271 $(M + H)^+$; Found = 304.2262 $(M + H)^+$, 326.2061 $(M + Na)^+$. ^1H NMR ($CDCl_3$, 400 MHz): δ_H = 7.50 (t, ^3J = 1.8 Hz, 1H), 7.25 (d, ^3J = 1.8 Hz, 2H), 3.85 – 3.40 (m, 8H), 1.35 (s, 18 H). ^{13}C NMR (101 MHz, $CDCl_3$) δ_C = ppm 171.37 (s, 1 C) 151.04 (s, 2 C) 134.56 (s, 1 C) 123.81 (s, 1 C) 121.18 (s, 2 C) 66.86 (s, 2 C) 53.44 (s, 2 C) 34.88 (s, 2 C) 31.35 (s, 6 C). Anal. Calcd for $C_{19}H_{29}NO_2$: C, 75.21; H, 9.63; N, 4.62. Found: C, 75.36; H, 9.74; N, 4.65.

1,9-bis(3,5-di-tert-butylbenzoyl)-5-(3,5-di-tert-butylphenyl)dipyrromethane. 3,5-di-*tert*-butylphenyl morpholin-4-yl ketone (2.28 g, 7.5 mmol) was added to a flame dried flask which had been backfilled with nitrogen three times. $POCl_3$ (1.4 mL, 15.0 mmol) was added and the mixture stirred at 65 °C for 3 h under a nitrogen atmosphere. A dark green solution

formed. The mixture was then cooled to room temperature, and toluene (50 mL) was added via canula. 3,5-di-*tert*-butylphenyl-substituted dipyrromethane (0.63 g, 1.9 mmol) was added in one portion, and the mixture was stirred at 90 °C for 2 h under a nitrogen atmosphere. The reaction was then quenched via addition of saturated NaOAc solution (100 mL) and stirred for a further hour at 90 °C. The mixture was cooled to room temperature and the organic layer separated. The aqueous layer was extracted with DCM (3 × 100 mL). The organic washings were combined, washed with water (3 × 100 mL), dried over $MgSO_4$ and evaporated under reduced pressure to yield crude product. This was purified via column chromatography (SiO_2, DCM -> DCM/2% EtOAc) to yield a dark purple solid (1.27 g, 88 %). MS (EI): m/z$^+$ Calc = 767.5510 $(M + H)^+$ Found = 767.5495 $(M + H)^+$, 789.5331 $(M + Na)^+$. ^1H NMR ($CDCl_3$, 400 MHz): δ_H = 9.58 (br. S., 2H), 7.73 (d, ^3J = 1.9 Hz, 4H), 7.64 (t, ^3J = 1.8 Hz, 2H), 7.42 (t, ^3J = 1.7 Hz, 1H), 7.17 (d, ^3J = 1.8 Hz, 2H), 6.80 (dd, ^3J = 3.8, 2.5 Hz, 2H), 6.16 (t, ^3J = 3.2 Hz, 2H), 5.61 (s, 1H), 1.39 (s, 36 H), 1.34 (s, 18 H). ^{13}C NMR ($CDCl_3$, 101 MHz) δ_C = 185.45 (s, 2 C) 151.37 (s, 2 C) 150.59 (s, 4 C) 140.49 (s, 2 C) 138.85 (s, 1 C) 138.00 (s, 2 C) 131.17 (s, 2 C) 125.68 (s, 2 C) 123.58 (s, 4 C) 122.97 (s, 2 C) 121.52 (s, 1 C) 120.40 (s, 2 C) 110.77 (s, 2 C) 45.21 (s, 1 C) 34.97 (s, 4 C) 34.93 (s, 2 C) 31.52 (s, 6 C) 31.46 (s, 12 C) ppm. Anal. Calcd for $C_{53}H_{70}N_2O_2$: C, 82.98; H, 9.20; N, 3.65. Found: C, 83.11; H, 9.28; N, 3.74.

1-formylphenyl-3-benzoyl-thymine. 3-benzoylthymine (2 g, 8.69 mmol), 4-formylphenylboronic acid (2.6 g, 17.4 mmol), $Cu(OAc)_2$ (2.37 g, 13.0 mmol) and molecular sieves (3 Å, 3 g) were added to a flame-dried flask. Dry DCM (70 mL) was added via canula, followed by the addition of pyridine (1.4 mL, 17.4 mmol) before the mixture was stirred at room temperature for 74 hours in air. Once a TLC showed that the reaction was complete, the mixture was diluted with DCM (50 mL), filtered through celite and the resulting green solution washed with water in the presence of EDTA (100 mg in 500 mL of distilled water). The organic phase was then dried over $MgSO_4$ and evaporated under reduced pressure to yield a cream coloured solid. This was purified via column chromatography (SiO_2, DCM -> DCM/5% EtOAc) to yield a white solid (2.48 g, 85%). MS (EI): m/z$^+$: Calc 357.0851 $(M + Na)^+$ Found 357.0841 $(M + Na)^+$. ^1H NMR ($CDCl_3$, 400 MHz): δ_H = 10.09 (s, 1H), 8.05 – 8.00 (m, 4H), 7.72 – 7.68 (m, 1H), 7.65 – 7.63 (m, 2H), 7.57 – 7.53 (m, 2H), 7.36 (d, ^3J = 1.1 Hz, 1H), 2.08 (s, 3H). ^{13}C NMR (101 MHz, $CDCl_3$) δ_C ppm = 190.72 (s, 1 C) 168.49 (s, 1 C) 162.84 (s, 1 C) 148.89

(s, 1 C) 143.00 (s, 1 C) 139.22 (s, 1 C) 135.97 (s, 1 C) 135.21 (s, 1 C) 131.48 (s, 1 C) 130.78 (s, 2 C) 130.51 (s, 2 C) 129.24 (s, 2 C) 126.81 (s, 2 C) 112.27 (s, 1 C) 12.49 (s, 1 C). Anal. Calcd for $C_{19}H_{14}N_2O_4$: C, 68.26; H, 4.22; N, 8.38. Found: C, 68.35; H, 4.16; N, 8.43.

1-phenyldipyrromethane-3-benzoylthymine. Freshly distilled pyrrole (15 mL, 210 mmol) and 1-formylphenyl-3-benzoyl-thymine (700 mg, 2.1 mmol) were added to a flask and degassed with nitrogen for 30 minutes. $InCl_3$ (93 mg, 0.42 mmol) was added and the mixture stirred for 2 h at room temperature under nitrogen. Once TLC indicated that all starting material had been consumed, the reaction was quenched via the addition of NaOH (167 mg, 4.2 mmol) and stirred at room temperature for 40 minutes. The solid impurities were removed via suction filtration and the solvent removed under reduced pressure to yield a dark brown foam. This was purified via column chromatography (SiO_2, DCM -> DCM/10% EtOAc) to yield a gold coloured solid (418 mg, 44 %). MS (EI): m/z^+: Calc 473.1590 $(M + Na)^+$ Found 473.1598 $(M + Na)^+$. ^1H NMR ($CDCl_3$, 400 MHz): δ_H = 8.06 – 7.99 (m, 4 H) 7.70 – 7.66 (m, 1 H) 7.55 – 7.51 (m, 2 H) 7.33 (br. s, 4 H) 7.31 – 7.30 (m, 1H), 6.72 (td, ^3J = 2.6 × 2, 1.6 Hz, 2 H), 6.18 (q, ^3J = 2.7 Hz, 2H), 5.93 – 5.91 (m, 2H), 5.52 (s, 1H), 2.04 (d, ^3J = 1.1 Hz, 3H). ^{13}C NMR (101 MHz, $CDCl_3$) δ_C ppm 168.84 (s, 1 C) 163.13 (s, 1 C) 149.42 (s, 1 C) 143.39 (s, 1 C) 140.40 (s, 1 C) 136.79 (s, 1 C) 135.10 (s, 1 C) 131.74 (s, 2 C) 131.63 (s, 1 C) 130.49 (s, 2 C) 129.62 (s, 2 C) 129.20 (s, 2 C) 126.37 (s, 2 C) 117.61 (s, 2 C) 111.34 (s, 1 C) 108.42 (s, 2 C) 107.42 (s, 2 C) 43.54 (s, 1 C) 12.40 (s, 1 C). Anal. Calcd for $C_{27}H_{22}N_4O_3$: C, 71.99; H, 4.92; N, 12.44. Found: C, 72.06; H, 5.00; N, 12.37.

Mono-phenyl(benzoylthymine)-tri-(3,5-di-tert-butylphenyl)porphyrin. 1,9-bis(3,5-di-*tert*-butylbenzoyl)-5-(3,5-di-*tert*-butylphenyl)dipyrromethane was first reduced to the corresponding dicarbinol. The diacyl compound (600 mg, 0.78 mmol) was dissolved in MeOH (14 mL) and THF (42 mL) and $NaBH_4$ (2.95 g, 78 mmol) was added in small portions over 30 minutes. During this process, the mixture changed colour from dark purple to orange. Water (30 mL) was then added to quench the reaction; the dicarbinol compound was then extracted with DCM, dried over K_2CO_3 and the solvent evaporated under reduced pressure. The resulting orange solid was dried under vacuum for 1 h before being carried on to the next step without purification.

The dicarbinol compound (0.78 mmol assuming previous step was quantitative) and 1-phenyldipyrromethane-3-benzoylthymine (350 mg, 0.78 mmol) were dissolved in MeCN (200 mL). TFA (0.7 mL, 9.36 mmol) was added and the mixture was stirred at room temperature for 5 minutes. DDQ (531 mg, 2.34 mmol) was added and the mixture was stirred at room temperature for 1 h. Et_3N (1.3 mL, 9.36 mmol) was added to neutralise the acid and the mixture was stirred at room temperature for 15 minutes. The resulting dark purple solution was filtered through alumina and the solvent evaporated under reduced pressure. The resulting dark purple residue was dissolved in DCM and passed through a pad of silica which was washed with DCM/10 % EtOAc. The solvent was evaporated under reduced pressure and the resulting purple residue purified via column chromatography (SiO_2, DCM) to yield pure product (160 mg, 17.5 %). MS (MALDI-TOF, DCTB Matrix, positive mode) Calc: 1178.68 Found: 1178.8 (M^+). ^{1}H NMR ($CDCl_3$, 400 MHz): δ_H = 8.94 – 8.91 (m, 6 H), 8.84 (d, ^3J = 4.8 Hz, 2H), 8.38 (d, ^3J = 8.5 Hz, 2 H), 8.15 – 8.12 (m, 2 H), 8.11 – 8.09 (m, 6 H), 7.84 – 7.82 (m, 5 H), 7.74 – 7.72 (m, 2 H), 7.63 – 7.59 (m, 2 H), 2.20 (d, ^3J = 1.2 Hz, 3H). Anal. Calcd for $C_{80}H_{86}N_6O_3$: C, 81.46; H, 7.35; N, 7.12. Found: C, 81.36; H, 7.46; N, 7.16. Single crystals suitable for analysis by X-ray diffraction were grown by slow diffusion of MeOH into a solution of the product in $CDCl_3$.

Mono-thymine-tri-(3,5-di-tert-butylphenyl)porphyrin; (mono-TP). Mono-benzoylthymine-tri-(3,5-di-*tert*-butylphenyl)porphyrin (100 mg, 0.08 mmol) was dissolved in THF (25 mL) and NH_4OH (10 mL). The mixture was stirred at room temperature for 18 h. The solvents were then removed under reduced pressure; the resulting purple residue was triturated with toluene and MeOH before being dried under vacuum overnight to yield a purple solid (84 mg, 92 %). MS (MALDI-TOF, DCTB matrix, positive mode) Calc: 1074.47 Found 1074.5 (M^+). ^{1}H NMR ($CDCl_3$, 400 MHz): δ_H = 8.91 - 8.99 (m, 6 H), 8.87 (d, ^3J = 4.7 Hz, 2 H), 8.39 (m(para), ^3J = 8.2 Hz, 2 H), 8.24 (s, 1 H), 8.12 (s, 4 H), 8.10 (d, ^3J = 1.8 Hz, 2 H), 7.81 - 7.87 (m, 3 H), 7.79 (m(para), ^3J = 8.4 Hz, 2 H) 2.16 (s, 3 H), -2.60 (br. s, 2 H). Anal. Calcd for $C_{73}H_{82}N_6O_2$: C, 81.53; H, 7.69; N, 7.81. Found: C, 81.59; H, 7.80; N 7.75. ^{13}C NMR (126MHz ,$CDCl_3$) δ_C = 171.2, 163.8, 150.2, 148.8, 148.7, 143.1, 141.2, 141.1, 140.9, 138.1, 135.4, 129.9, 129.7, 124.4, 121.9, 121.6, 121.1, 117.5, 111.6, 35.1, 31.8, 21.1. Single crystals suitable for analysis by X-ray diffraction were grown by slow diffusion of MeOH into a solution of the product in CH_2Cl_2.

Tetra-(phenyl-(3-benzoylthymine))porphyrin. 1-formylphenyl-3-benzoyl-thymine (1 g, 2.99 mmol) was added to a flame-dried flask which had been backfilled with nitrogen three times. DCM (300 mL) was added via canula and freshly distilled pyrrole (0.21 mL, 2.99 mmol) was added via syringe. TFA (1 mL, 5.7 mmol) was added and the reaction mixture stirred for 5 minutes at room temperature, during which time a colour change from pale yellow to dark purple was observed. DDQ (678 mg, 2.99 mmol) was added and the mixture stirred at room temperature for 1 h. TLC analysis indicated that porphryin formation had occurred. Et_3N (1 mL, 13.6 mmol) was added and the mixture stirred at room temperature for 15 minutes. The purple solution was then filtered through alumina and evaporated under reduced pressure to yield a purple residue. The residue was purified via column chromatography (SiO_2, DCM/5% EtOAc -> DCM/10% EtOAc) to yield a purple powder (124 mg, 11 %). MS (MALDI-TOF, DCTB matrix, positive mode) Calc: 1526.46 (M^+) Found 1526.5 (M^+). ^1H NMR (400 MHz, $CDCl_3$) δ_H ppm 8.87 (s, 8 H), 8.34 (d, ^3J = 8.4 Hz, 8 H), 8.15 - 8.12 (m, 8 H), 7.85 (d, ^3J = 8.4 Hz, 8 H) 7.76 – 7.73 (m, 8 H), 7.63 - 7.60 (m, 8 H) 2.21 (m, 12 H), -2.83 (s, 2 H). Anal. Calcd for $C_{92}H_{62}N_{12}O_{12}$: C, 72.34; H, 4.09; N, 11.00. Found: C, 72.24; H, 4.18; N, 10.89.

Tetra-(phenylthymine)porphyrin; (tetra-TP). Tetra-(phenyl-(3-benzoylthymine))porphyrin (100 mg, 0.07 mmol) was dissolved in THF (100 mL) and NH_4OH (25 mL) and stirred at room temperature for 22 h. The liquids were then decanted and the remaining residue dried under vacuum before being triturated with toluene and MeOH. The purple solid was then suspended in MeOH, isolated via centrifugation and dried under vacuum to yield a purple powder (70 mg, 91 %). MS (MALDI-TOF, DCTB matrix, positive mode) Calc: 1110.37 (M^+) Found: 1110.4 (M^+). ^1H NMR (400 MHz, $CDCl_3$ + TFA-d, protonated porphyrin) δ_H ppm 8.88 (s, 8 H), 8.76 (d, ^3J = 8.4 Hz, 8 H), 8.13 (d, ^3J = 8.2 Hz, 8 H) 7.81 (s, 4 H), 2.24 (s, 12 H).

Single crystal X-ray diffraction Studies:

Single crystal diffraction data of mono-TP and benzoyl-mono-TP were collected at 120(2)K on either Beamline I19 at Diamond Light Source (DLS-I19)[S1] (mono-TP) or an Oxford Diffraction SuperNova using mirror monochromated Cu-Kα radiation (benzoyl-mono-TP). Using Olex2[S2], the structure was solved with the Superflip[S3] structure solution program using

Charge Flipping and refined with the ShelXL[S4] refinement package using Least Squares minimisation.

For the refinement of the structure of mono-TP the *tert*-butyl group C41A-C44B is disordered over two orientations. Equivalent 1,2- and 1,3-distances of the disordered methyls were restrained to be approximately equal. PLATON SQUEEZE was applied to the data to remove the scattering contribution from a disordered MeOH solvent molecule which could not be sensibly modelled and produce a set of solvent-free diffraction intensities for the final cycles of refinement. A total of 20 electrons were removed from the unit cell, equating to approximately 1 MeOH molecule per formula unit: these were included in the unit cell contents.

For the refinement of the structure of benzoyl-mono-TP geometric similarity restraints were applied to the 1,2 and 1,3 distances of all *tert*-butyl groups and $CHCl_3$ solvent molecules (SAME). Rigid bond and similarity restraints were applied to the anisotropic thermal displacement parameters of all atoms in the structure (RIGU, SIMU). Orientational disorder was modelled in $CHCl_3$ molecule C1C/C1D with their occupancies refined with a free variable (C1C occupancy 0.49(1)) and constrained to sum to unity. The locations of the pyrrolic nitrogen bound hydrogen atoms of the porphyrin ring could not be identified in an electron difference map and so were placed geometrically and refined using a riding model at half occupancy as disordered pairs of opposing positions. PLATON SQUEEZE was applied to the data to remove the scattering contribution from several disordered solvent moieties and produce a set of solvent-free diffraction intensities for the final cycles of refinement. A total of 175 electrons were removed from the unit cell, equating to approximately 1.5 $CHCl_3$ molecules per formula unit: these were included in the unit cell contents.

Crystal Data for mono-TP; $C_{74}H_{86}N_6O_3$ (M =1107.48): triclinic, space group P-1 (no. 2), a = 9.8270(6) Å, b = 16.9659(8) Å, c = 21.6298(13) Å, α = 69.273(5)°, β = 81.505(5)°, γ = 87.976(5)°, V = 3335.2(4) $Å^3$, Z = 2, T = 120(2) K, μ(Synchrotron) = 0.063 mm^{-1}, $Dcalc$ = 1.103 g/mm^3, 31197 reflections measured, 11500 unique (R_{int} = 0.0549) which were used in all calculations. The final R_1 was 0.0756 ($I > 2\sigma(I)$) and wR_2 was 0.2097 (all data).

Crystal Data for Mono-phenyl(benzoylthymine)-tri-(3,5-di-tert-butylphenyl)porphyrin.6 $CHCl_3$; $C_{86}H_{92}Cl_{18}N_6O_3$ (M = 1895.75): triclinic, space group P-1 (no. 2), a = 15.4721(5) Å, b = 16.3717(5)) Å, c = 19.0278(6)Å, α = 92.068(2) °, β =110.731(3) °, γ = 99.280(3)°, V = 4426.0(3) Å^3, Z = 2, T = 120(2) K, μ(Cu-Kα) = 0.063 mm^{-1}, $Dcalc$ = 1.422 g/mm^3, 52160 reflections measured, 15638 unique (R_{int} = 0.079) which were used in all calculations. The final R_1 was 0.1220 (I > 2σ(I)) and wR_2 was 0.3846 (all data).

Binding Studies:

Deuterated chloroform was filtered through activated basic alumina and dried over 4Å molecular sieves. Dilution and titration experiments was repeated twice, reported error are twice the standard error.

Self-association studies were performed separately for mono-TP and 9-propyladenine using the following approach. A high concentration of analyte was prepared in $CDCl_3$ and 450 µl this solution was transferred to a capped NMR tube. A small volume of pure $CDCl_3$ was added successively to dilute the sample and ^{1}H NMR spectra recorded following each dilution. The data was fitted to dimerisation model by solving the equations below using non-linear curve fitting procedure.[S5] In each case the NH chemical shift was used for fitting using the following equations.

$$\mathrm{A} + \mathrm{A} \rightleftharpoons \mathrm{AA}$$

$$[\mathrm{AA}] = \frac{1 + 4K_d[\mathrm{A}]_0 - \sqrt{\{1 + 8K_d[\mathrm{A}]_0\}}}{8K_d}$$

$$[\mathrm{A}] = [\mathrm{A}]_0 - 2[\mathrm{AA}]$$

$$\delta_{\mathrm{obs}} = \frac{2[\mathrm{AA}]}{[\mathrm{A}]_0}\delta_{\mathrm{d}} + \frac{[\mathrm{A}]}{[\mathrm{A}]_0}\delta_{\mathrm{f}}$$

Where $[A]_0$ is the total concentration, [A] is the concentration of the unbound free species, [AA] is the concentration of the dimer, K_d is the dimerization constant, δ_f is the free chemical shift and δ_f is the limiting bound shift of the dimer.

Mono-TP: $K_d = 6.1 \pm 3.0\ M^{-1}$

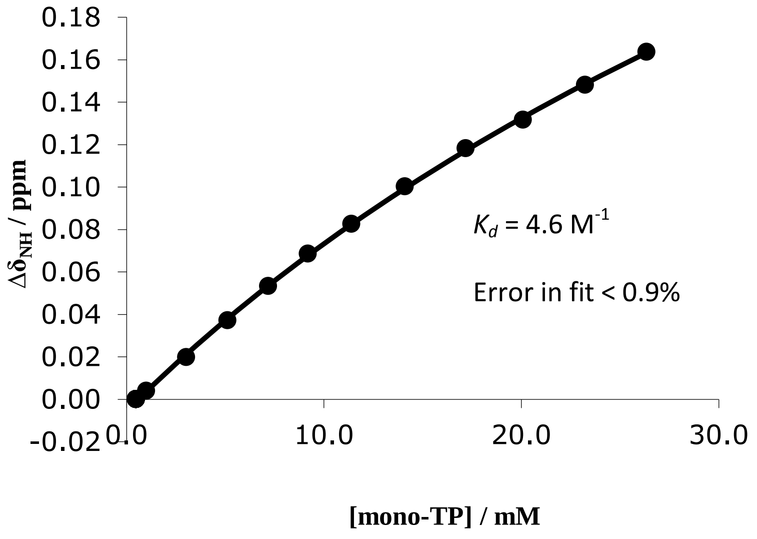

Fig. S1: Plot of Mono-TP concentration vs. shift in the thymine N*H* ^{1}H NMR peak.

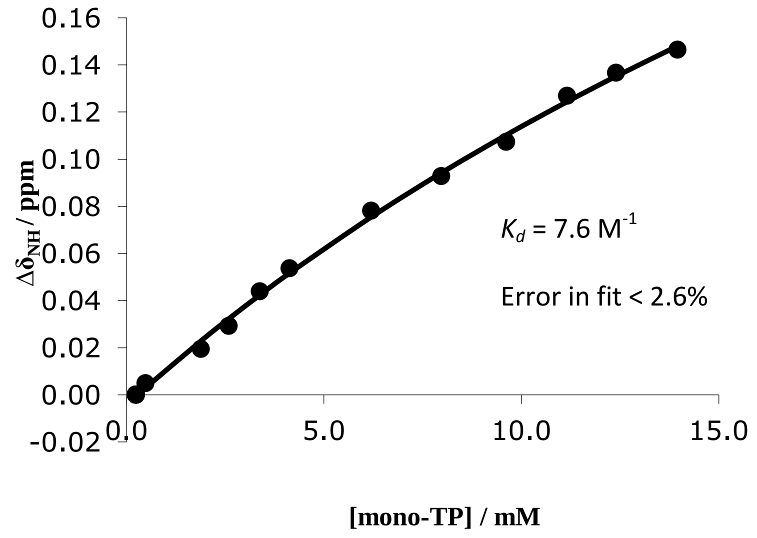

Fig. S2: Plot of Mono-TP concentration vs. shift in the thymine N*H* ^{1}H NMR peak.

9-propyladenine: K_d = 2.8 ± 1.7 M^{-1}

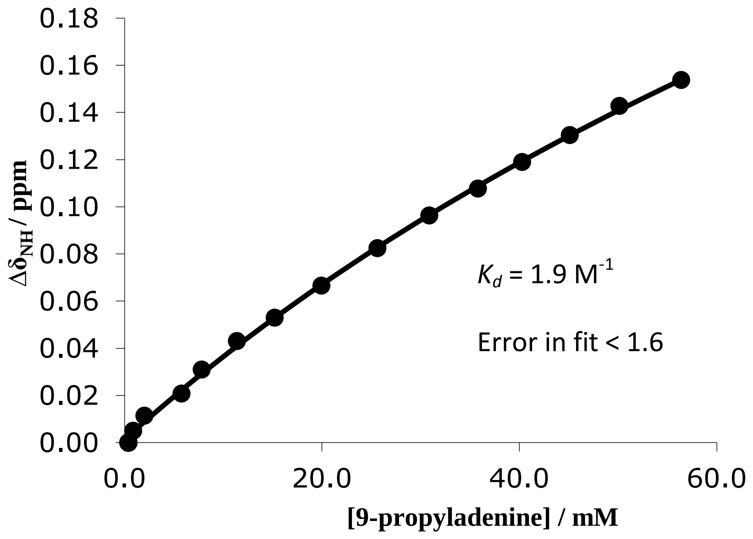

Fig. S3: Plot of 9-propyladenine concentration vs. shift in the adenine N*H* ^{1}H NMR peak.

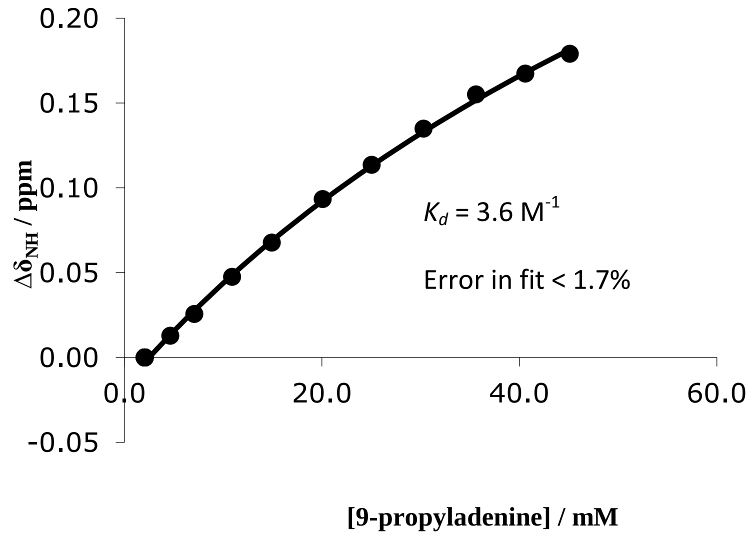

Fig. S4: Plot of 9-propyladenine concentration vs. shift in the adenine N*H* ^{1}H NMR peak.

Mono-TP / 9-propyladenine Titration: $K = 91.8 \pm 20.5\ M^{-1}$

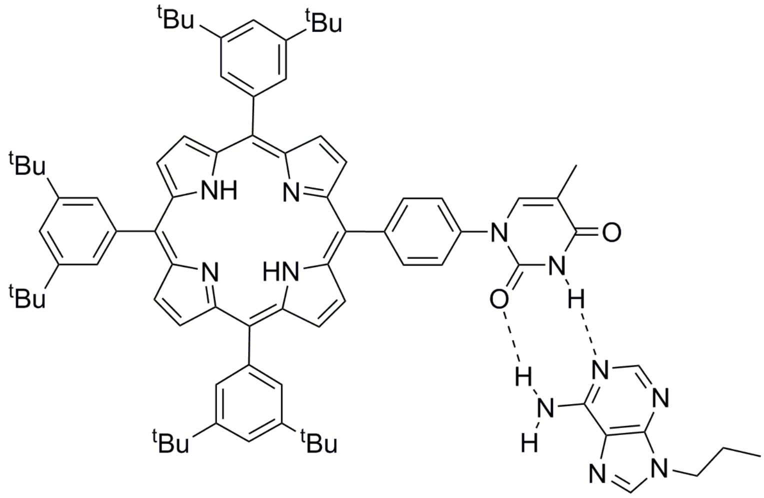

Mono-TP was employed as the host and the solution concentration was kept constant. Mono-TP was dissolved in $CDCl_3$ to make a 2 ml solution (4 – 5 mM). 500 µl of the mono-TP solution was transferred to a capped NMR tube and a ^{1}H NMR spectrum was recorded. The 9-propyladenine compound was dissolved in the remaining host solution and titrated into the NMR tube. ^{1}H NMR spectra were recorded following addition of each titre. The data was fitted to 1:1 binding isotherm by solving the equation below using non-linear curve fitting procedure.[S6] The Chemical shift of mono-TP N*H* on host was used for fitting using the following equations.

$$\mathrm{H} + \mathrm{G} \rightleftharpoons \mathrm{HG}$$

$$[\mathrm{HG}] = \frac{1 + K[\mathrm{H}]_0[\mathrm{G}]_0 - \sqrt{\{(1 + [\mathrm{H}]_0[\mathrm{G}]_0)^2 - 4\,K^2[\mathrm{H}]_0[\mathrm{G}]_0\}}}{2K}$$

$$[\mathrm{H}] = [\mathrm{H}]_0 - [\mathrm{HG}]$$

$$\delta_{\mathrm{obs}} = \frac{[\mathrm{HG}]}{[\mathrm{H}]_0}\delta_{\mathrm{b}} + \frac{[\mathrm{H}]}{[\mathrm{H}]_0}\delta_{\mathrm{f}}$$

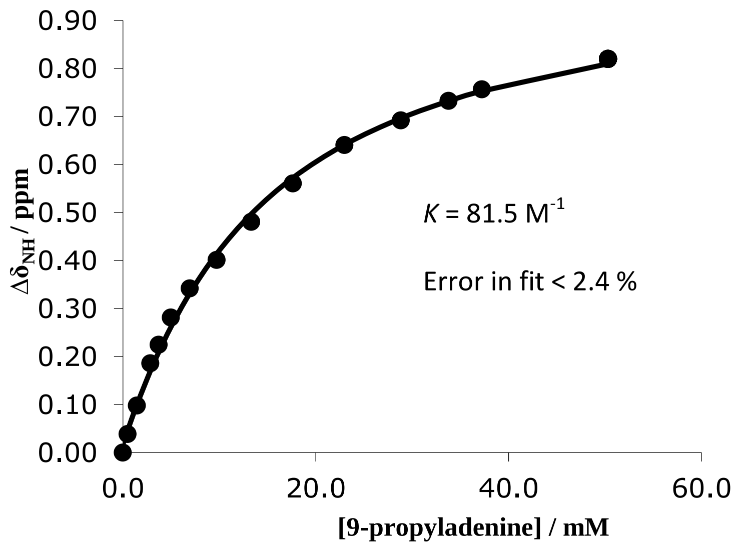

Fig. S5: Plot of 9-propyladenine concentration vs. shift in the mono-TP thymine N*H* ^{1}H NMR peak.

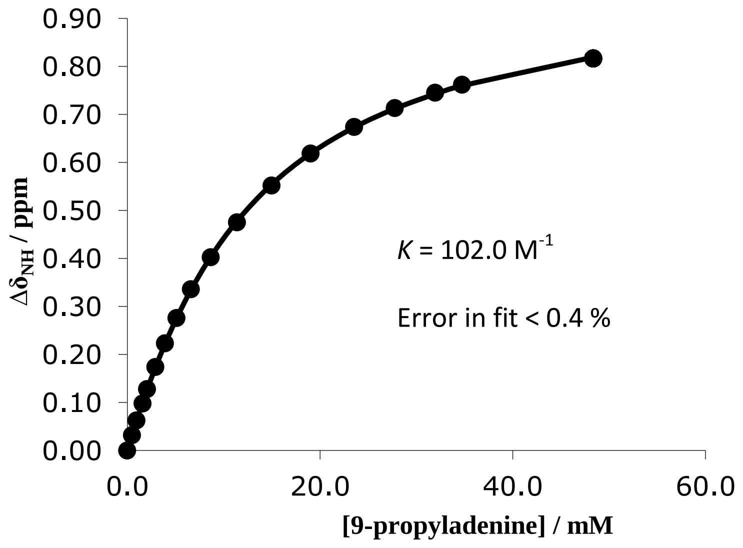

Fig. S6: Plot of 9-propyladenine concentration vs. shift in the mono-TP thymine N*H* ^{1}H NMR peak.

STM Studies:

Tetra- TP was dissolved in a 1:9 volume ratio mixture of tetrahydrofuran (THF) (Sigma Aldrich, anhydrous, >99.9%) and 1,2,4-trichlorobenzene (TCB) (Sigma Aldrich, anhydrous, >99.9%) producing a 3.5 $\times 10^{-5}$ M solution. 9-propyladenine was dissolved in TCB at a concentration of 2.3×10^{-3} M and used as a stock solution to produce a range of TTP/9-propyladenine mixtures with various molar ratios and overall molar concentrations.

STM samples were produced by mounting a freshly cleaved HOPG sample (Agar Scientific) in a PTFE liquid-cell and pre-heating the substrate to 60°C using a sample heating stage positioned beneath the substrate. The liquid cell was then filled with ~50μL of the desired solution while the substrate was maintained at 60°C. After 10 minutes of heating at 60°C, the samples were allowed to cool naturally before being loaded in to the STM for imaging. STM experiments were carried out directly at the liquid-solid interface between the TCB/THF mixture and HOPG using a 5500 series SPM system (Keysight Technologies) operating under ambient conditions and at room temperature. The STM tips were mechanically cut from Pt/Ir (80:20) wire (Advent Research Materials Ltd). All images were obtained in constant current mode and have had a flattening procedure applied to them.

Drift correction of high resolution STM images was carried out using the Scanning Probe Image Processor (SPIP) software (Image Metrology ApS, Lyngby, Denmark). This process involved the collection of a high resolution image of a molecular structure immediately followed by collecting an image of the underlying HOPG lattice. The image of the HOPG lattice was collected using identical scanning parameters apart from the tunnel current and bias voltage. By assuming that the level of drift is constant for both images the known lattice dimensions of HOPG can be used to produce a set of correction parameters for the HOPG image. These parameters can them be applied to the high resolution molecular image to produce a drift corrected image. Figure S7 shows a pair of drift corrected images showing both the molecular (left) and HOPG (right) images. All unit cell dimensions and angles are obtained by taking the mean of at least three measurements taken from separate drift-corrected STM images. The stated error values for these measurements are the standard deviation of the various measurements. Fig. S8 shows two drift corrected STM images that demonstrate the chirality of the tetra-TP structure. These images show the two possible

mirror image domains for the tetra-TP network formed at the HOPG-TCB/THF solid-liquid interface.

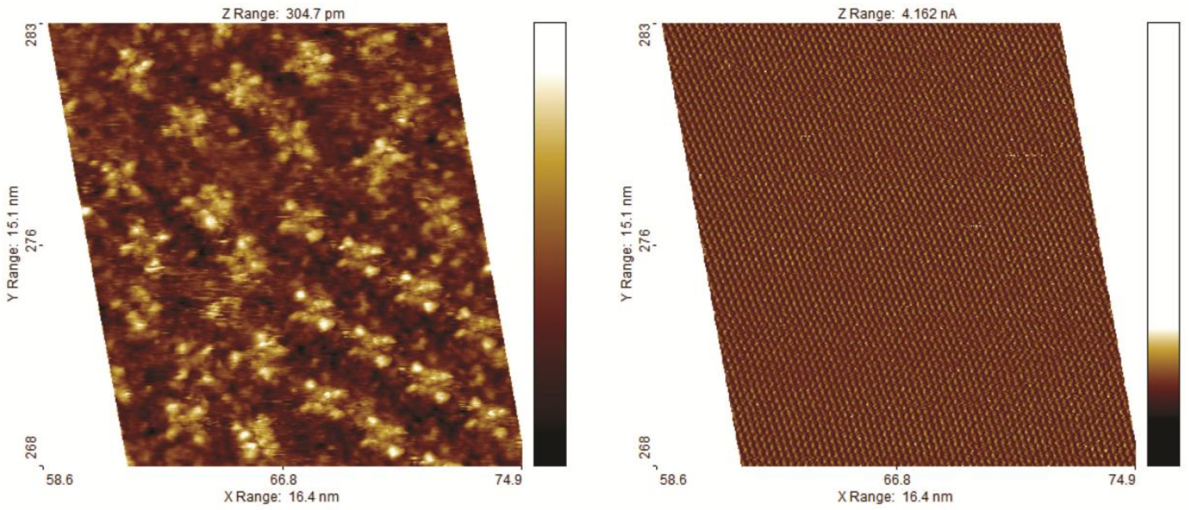

Fig. S7: A pair of drift corrected STM images for the tetra-TP and 9-propyladenine network showing the molecular network (left) and the underlying HOPG (right). STM imaging parameters: left V_s= -0.5 V; I_t= 20 pA and right V_s= -0.05 V; I_t= 1 nA.

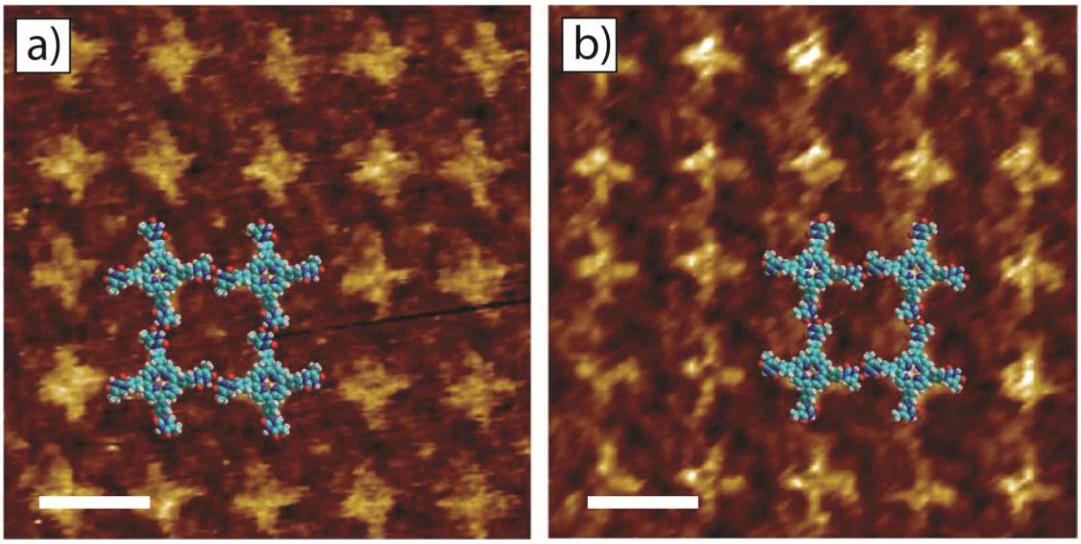

Fig. S8: STM images of the tetra-TP network (2.82 ×10^{-5} M) at the HOPG-TCB/THF solid-liquid interface. The images show the domains of the tetra-TP elf-assembled network with different chirality. Both images have been drift corrected. STM imaging parameters: V_s= -1.3 V; I_t= 20 pA. Scale bars: 27 Å.

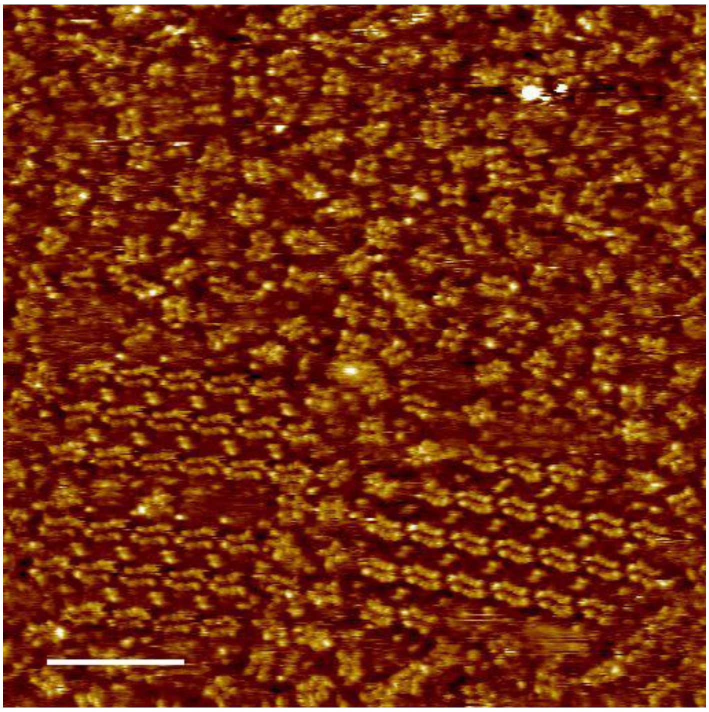

Fig. S9: An STM image of the tetra-TP (9.59×10^{-5} M) and 9-propyladenine (2.05×10^{-4} M) network showing domains with different chirality growing at the HOPG-TCB/THF solid-liquid interface. STM imaging parameters: V_s= -0.55 V; I_t= 15 pA. Scale bar: 100 Å.

In an analogous manner to the tetra-TP network, the tetra-TP and 9-propyladenine co-crystal also forms homochiral domains. Within a single domain all of the thymine groups on the tetra-TP molecule adopt the same orientation with respect to the porphyrin core. This separation of the prochiral tetra-TP molecules produces two possible mirror image domain structures for the tetra-TP and 9-propyladenine co-crystal, Fig. S9. An example of the molecular arrangement for one of these mirror image structures is given in Fig. S10b.

Molecular Mechanics (MM) Studies:

MM simulations of the tetra-TP and tetra-TP/9-propyladenine surface structures were carried out using the HyperChem software package. A single layer of graphite was fixed in place and used as a substrate. On top of this layer were placed the molecules representing an individual unit cell of the different 2D structures. The starting positions for the molecules were derived using drift-corrected STM images as a guide and maintaining a 0.35 nm vertical distance between the planar porphyrin and adenine cores of the molecules and the

underlying graphite layer. Once positioned, these structures were geometry optimised using the MM+ force field. The optimisation process was terminated when a gradient < 0.01 kcal $Å^{-1}$ mol^{-1} was reached. All unit cell dimensions and angles are taken from geometry optimised structures. Fig. S10 shows the geometry optimised molecular structures with the underlying layer of graphite: tetra-TP network (Fig. S10a) and the tetra-TP/9-propyladenine co-crystal (Fig. S10b).

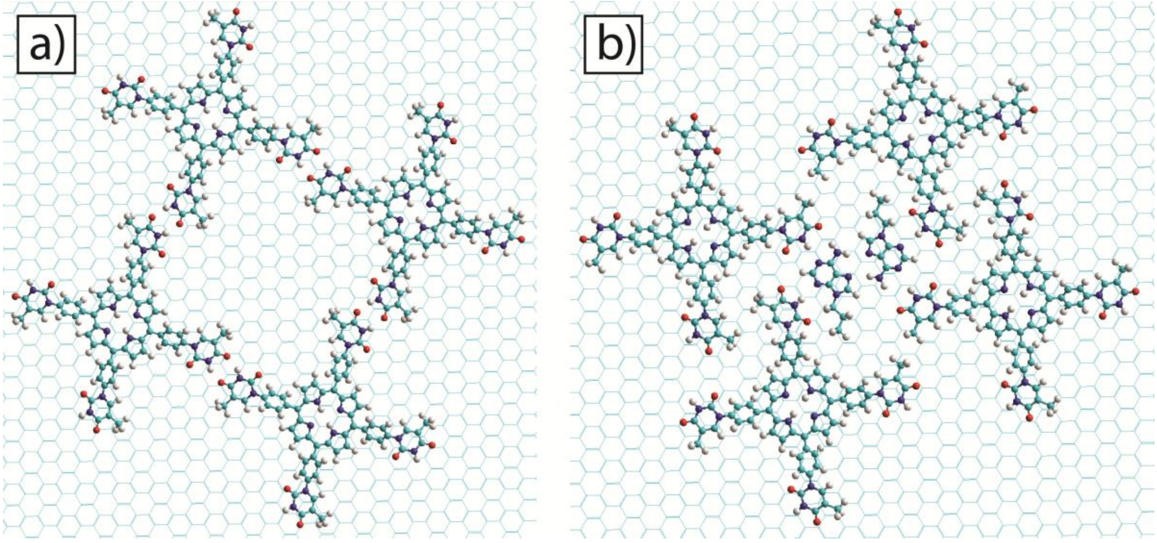

Fig. S10: Geometry optimised MM simulations of **a)** the tetra-TP network; and **b)** the tetra-TP and 9-propyladenine co-crystal.

References.

S1. H. Nowell, S. A. Barnett, K. E. Christensen, S. J. Teat and D. R. Allan, *J. Synchrotron Rad.* 2012, **19**, 435-441.

S2. O. V. Dolomanov, L. J. Bourhis, R. J. Gildea, J. A. K. Howard and H. Puschmann, *J. Appl. Cryst*. 2009, **42**, 339-341.

S3. SUPERFLIP, *J. Appl. Cryst*., 2007, **40**, 786-790.

S4. G.M. Sheldrick, *Acta Cryst*., 2008, **A64**, 112-122.

S5. A. P. Bisson, F. J. Carver, D. S. Eggleston, R. C. Haltiwanger, C. A. Hunter, D. L. Livingstone, J. F. McCabe, C. Rotger and A. E.Rowan, *J. Am. Chem. Soc.,* 2000**,** **122**, 8856-8868.

S6. A. P. Bisson, C. A. Hunter, J. C. Morales and K. Young, *Chem. Eur. J.*, 1998**, 4**, 845-851.
